# When need for closure leads to positive attitudes towards a negatively stereotyped outgroup

**DOI:** 10.1007/s11031-014-9414-5

**Published:** 2014-06-01

**Authors:** Małgorzata Kossowska, Piotr Dragon, Marcin Bukowski

**Affiliations:** Institute of Psychology, Jagiellonian University, Al. Mickiewicza 3, 31-120 Kraków, Poland

**Keywords:** Need for closure, Ability to achieve closure, Stereotyping, Attitudes towards outgroup

## Abstract

The study examined the relationship between epistemic motivation, which is the need for closure (NFC), and positive attitudes towards a negatively stereotyped outgroup (i.e., Gypsies). Although extensive research has revealed that NFC is related to derogatory behavioural tendencies and negative emotions towards stereotyped groups, it is proposed that NFC may also be linked to positive attitudes towards outgroups. It is predicted, however, that this would be true only when NFC is accompanied by a low ability to achieve closure (AAC). It is argued that low AAC impairs the construction of schema and their effective application. Therefore, NFC in individuals with low AAC may lead them to correct their initial tendency to use stereotypes and, as a consequence, to evaluate a negatively stereotyped outgroup in a positive way. In this research, low AAC was assessed by a scale (Study 1) and experimentally induced (Study 2). In both studies, we measured positive attitudes towards Gypsies. The results of the studies supported our prediction that NFC is positively related to positive attitudes towards Gypsies when AAC is low.

## Introduction

In recent years, the motivated social cognition perspective has tried to explain people’s beliefs and attitudes towards different groups. In particular, in his work on lay epistemics, Kruglanski (1989) has argued that stereotyping and prejudice stem from particular motivational needs, rather than being merely a cognitive deficit. An important concept in this approach is the need for closure (NFC), which refers to the individual’s desire for firm answers and their aversion to ambiguity (Kruglanski 1989). As such, the NFC has been described as the tendency to reduce the feeling of discomfort experienced in the face of cognitive uncertainty, through the quick formulation and validation of a hypothesis (Webster and Kruglanski 1994). It is well documented that cognitive processes that are used by individuals with high NFC to reduce uncertainty are category-based, non-systematic, and heuristic (for a review see, Kruglanski et al. 2009). In contrast, individuals with a low NFC prefer to reduce uncertainty by using piecemeal or individuation processes. This latter preference is manifested in vigilant behaviour that is based on a systematic and effortful search, evaluation, and unbiased assimilation of relevant information (Bar-Tal et al. 1997; Driscoll et al. 1991; Kruglanski et al. 2009).

### Need for closure and attitudes towards outgroups

The NFC has been found to affect a broad range of social cognitive processes, including group processes and intergroup attitudes. Many studies have demonstrated that because the NFC fosters a desire for firm knowledge, and that such knowledge is grounded in the shared reality of one’s group, NFC also fosters a greater affinity for one’s ingroup than for outgroups (e.g., Golec and Federico 2004; Kruglanski et al. 2002; Kruglanski and Webster 1996; Shah et al. 1998). In addition, people with a higher NFC have been found to be more motivated to rely on group consensus to guide their judgments, compared to people with a lower NFC (Kruglanski et al. 2006). Thus, when the socially shared stereotype of the outgroup was strongly negative, high NFC individuals tended to develop more negative attitudes towards the outgroup than did low NFC individuals. It is also worth mentioning that various studies of NFC have focused on its influence on authoritarianism, essentialism, political conservatism, and prejudice (e.g., Roets and Van Hiel 2011; Van Hiel et al. 2004).

### Need for closure and ability to achieve closure as predictors of attitudes towards outgroups

Recent studies however have provided evidence that the main effects of NFC on the aforementioned variables may be substantially qualified by an interaction with the ability to achieve closure (AAC; Bar-Tal and Guinote 2002; Bar-Tal et al. 1997, 1999; Kossowska and Bar-Tal 2013a; Otten and Bar-Tal 2002). The level of AAC is defined as the extent to which individuals expect to be able to use cognitive schema (e.g., stereotypes) effectively to form a judgment. Thus, in a situation of high AAC, the NFC may increase the use of heuristic processing or stereotyping. However, in a situation of low AAC, the NFC may not be related to construct and apply schema effectively, thus may lead to systematic or individuating processing. Indeed, Kossowska and Bar-Tal (2013a) demonstrated that in high-AAC individuals, NFC is directly related to stereotypical thinking and negative attitudes toward the outgroup, but in low-AAC individuals, NFC is inversely related to stereotypical thinking and negative attitudes toward the outgroup.

In the present study, we have focused on positive attitudes towards outgroups, which have been largely neglected in previous studies. We believe that although it is critical to understand and reduce negative attitudes, it is also critical to understand and promote positive attitudes (see Pittinsky 2009; Pittinsky et al. 2011a, b). There is some evidence that positive and negative attitudes should be treated as separate dimensions (e.g., Cacioppo and Berntson 1994; Pittinsky et al. 2011b) because they are nonreciprocally activated. That is, a change in a negative attitude does not necessarily coincide with an equivalently countervailing change in the corresponding positive attitude (Cacioppo et al. 1999). These functionally separate positive and negative attitude–behaviour links can occur automatically (Chen and Bargh 1999) and they are evident in the different neurological systems involved in the assessment of a stimulus as friendly or hostile (LeDoux 1995). Since positive attitudes may change at different times and rates than negative attitudes, the current level of a positive attitude cannot be inferred from the reverse of a score on a measure of a current negative attitude (Cacioppo and Berntson 1994; Cacioppo et al. 1997, 1999).

Taking these findings into account, in this paper we have focused on the degree to which the NFC influences positive attitudes towards an outgroup. Although previous research suggested that NFC is related to negative attitudes towards outgroups, we propose that this may be true only among high AAC people. Among low AAC people, we hypothesized that NFC is positively associated with positive attitudes towards outgroups. We explain this assumption in detail below.

### When NFC is related to positive attitudes toward negatively stereotyped outgroups

Previous research revealed that in low-AAC individuals, NFC is related to the low use of heuristic processing styles (Bar-Tal et al. 1997; Kossowska and Bar-Tal 2013a). It is also suggested that this low-level use of heuristic processing may not be a manifestation of a controlled and wilful cognitive process that is aimed at reaching a valid conclusion (as vigilance is), but rather, that it may be a manifestation of being helpless to construct a schema and apply it effectively (see also Guinote 2007; Sedek and Kofta 1990). In other words, if individuals are unable to construct and apply negative stereotypes, they may tend to alter their initial tendency to use stereotypes and, as a result, they may use more systematic processing style, i.e., they may be less sensitive to information relevancy and attend to and examine of more information. Thus, in their evaluation of a negatively stereotyped outgroup, for low AAC people, NFC would be related to more awareness of positive information about the stereotyped group and develop more positive or balanced perception of the outgroup.

It is worth emphasising that we did not make any predictions about the relationship between NFC and positive attitudes towards outgroup among high AAC individuals. We expected that in high AAC individuals, NFC is related to the effective use of schematic knowledge, thus it would lead to a negative evaluation of members of negatively perceived groups. Thus, as some studies have demonstrated, the effect of NFC among high AAC individuals applies to negative stereotypes, and negative, but not positive, attitudes (see: Bar-Tal et al. 1997; Kossowska and Bar-Tal 2013a; Otten and Bar-Tal 2002).

### Overview of the study

In both studies, we assessed the levels of NFC as well as positive attitudes towards the target group. On the basis of previous studies (e.g., Bukowski and Winiewski 2011; Strzeszewski 2003), Gypsies were chosen as a group towards which Poles hold negative stereotypes and are prejudiced against. In Study 1 we measured AAC as an individual characteristic. In Study 2 we used informational helplessness training to operationalize low AAC. Sedek and Kofta (1990) demonstrated that informational helplessness decreases the effective use of schemata. Thus, we expected that a strong experience of informational helplessness (i.e., repeated failure on a task that requires cognitive structuring) would reduce AAC. We hypothesized that NFC may lead to positive attitudes to outgroups among low AAC people. This hypothesis is based on the assumption that NFC is linked to the motivation to use stereotypical thinking and develop negative attitudes towards outgroups among high AAC people. However, low AAC leads to an inability to finish the epistemic process sufficiently to avoid inconsistent information, to form and use unequivocal, one-sided, unambiguous categorisations or to apply the schema efficiently. Thus, under low AAC, NFC leads to an alteration of the initial tendency to use schematic knowledge, and to process information that is inconsistent with the previous knowledge (i.e., positive). As a consequence, NFC leads to the development of positive attitudes toward the negatively stereotyped outgroups.

## Study 1

In Study 1, we tested the hypothesis that, depending on their NFC, the degree to which the respondents develop positive attitudes towards the target group would be differentially affected by their AAC. Our premise was that among individuals with low AAC, NFC is related to avoidance of negative evaluations and the tendency to foster positive evaluations. To make our argument about the separate mechanisms of positive and negative attitudes stronger, we also measured negative stereotypes that participants used to create their impressions of outgroup members. We hypothesised that NFC is related to negative stereotype usage among high (but not low) AAC individuals.

### Method

#### Participants

The study was conducted online and links to the survey^1^ were distributed through popular Polish portals to recruit participants. Five hundred and nineteen internet users (262 men, 257 women) completed all the measures in the survey. The age of the participants ranged from 17 to 27 years of age (*M* = 21.42, SD = 1.65). Of the respondents, 27.0 % had completed higher education, 65.2 % had completed secondary education, and 17.8 % left school before completing secondary education. The survey respondents received 30 PLN (approximately 5 Euro) for participating in the survey.

#### Materials and procedure

Participants completed a short version of the NFC Scale (Kossowska et al. 2012) at the beginning of the session. We used four of the five subscales of the NFC scale (Webster and Kruglanski 1994): preference for order and structure in the environment, predictability of future contexts, affective discomfort occasioned by ambiguity, and closed-mindedness. We excluded the decisiveness subscale because it is has been found to measure ability rather than motivation (Bar-Tal and Kossowska 2010; Roets and Van Hiel 2007). Respondents rated 12 items on a six-point scale (from 1 = completely disagree to 6 = completely agree). The mean score for all items was calculated (Cronbach’s *α* = .84, *M* = 3.9, SD = 0.72). A higher mean score indicated a higher NFC.

The ability to reach closure was measured by a six-point scale (from 1 = completely disagree to 6 = completely agree) that consisted of nine items representing high and low AAC. An example of an item demonstrating high ability is: “I do not bother with simple matters, I usually know what to do at once”. An example of an item demonstrating low ability is: “I tend to postpone important decisions to the last moment, and even then I have problems making them”. The construction and validation of the scale is described in Bar-Tal and Kossowska (2010). In the present study, the overall mean score of all items was calculated (Cronbach’s *α* = .79, *M* = 3.60, SD = 0.80). A higher score on the scale implied a higher AAC.

To measure positive attitudes towards Gypsies, participants completed a 10-item scale that was based on the Allophilia Scale by Pittinsky et al. (2011a). We used items measuring positive affective evaluations of outgroup members (“In general, I have positive attitudes about Gypsies”; “I feel positively towards Gypsies”), feelings of ease with outgroup members (“I find it easy to talk to Gypsies”; “There is no awkwardness for me when I’m around Gypsies”), tendency to seek opportunities to affiliate and interact with outgroup members (“I am truly interested in understanding the points of view of Gypsies”; “I am motivated to get to know Gypsies”), and having emotionally heightened positive attitudes about outgroup members (“I admire the Gypsy community members who live here under difficult circumstances”; “I am impressed by Gypsies”; “I feel inspired by Gypsies”; “I am enthusiastic about Gypsies”). Participants expressed agreement on a six-point scale (from 1 = completely do not agree to 6 = completely agree). We calculated scores for positive attitudes towards outgroup members (*M* = 3.50; SD = 0.67). The reliability of the scale achieved an acceptable level of internal consistency (Cronbach’s α = .79). We also used three items proposed by Craig and Richeson (2012) to measure the participants’ perceived closeness to Gypsies. Respondents were asked to indicate how close they felt their ideas, interests, and feelings were to those of Gypsies on a nine-point scale (1 = not close at all to 9 = very close) (Cronbach’s α = .90). Higher scores indicate greater perceived closeness to the group (*M* = 5.61; SD = 1.43).

In addition, we used a list of 13 stereotypical characteristics,^2^ which was tested in a previous study by Kofta and Narkiewicz-Jodko (2003) that asked participants to assess to what extent they agreed that typical Gypsies had these characteristics (Cronbach’s α = .77; *M* = 5.20; SD = 0.59). We calculated the negative stereotype index from these assessments.

### Results

Gender did not significantly predict any outcomes in the study and, therefore, it was excluded from the analyses.

NFC was correlated with the stereotype index (*r* = .25, *p* = .01) and perceived closeness (*r* = −.14, *p* = .01), but not with positive attitudes towards Gypsies (*r* = .03, *p* = .49) or AAC (*r* = −.01, *p* = .89). AAC was not significantly correlated with any of the dependent measures.

To examine the moderating effect of AAC, two-step hierarchical regression analyses were performed on the dependent measures. NFC and AAC were centred and entered into the first regression model. The interaction term (NFC × AAC) was added in the second model. The results of the analyses revealed a significant interaction between NFC and AAC that predicted the stereotype index (*R* ^2^ = .03; *F*(1,515) = 6.85; *p* = .01) and perceived closeness towards Gypsies (*R* ^2^ = .04; *F*(1,515) = 9.08; *p* = .01). The interaction of NFC and AAC approached statistical significance for positive attitudes towards Gypsies (*R* ^2^ = .03; *F*(1,515) = 3.61; *p* = .06; see Table 1).

**Table 1:** Regression coefficients (Study 1)

Dependent variables	Unstandardised coefficients		Standardised coefficients	t	Sig.	Adj. *R* ^2^
	B	SE	Beta			
*Perceived closeness*						
(Constant)	5.61	0.06		90.73	.01	.03
NFC	−0.22	0.06	−0.15	3.56	.01	
AAC	0.01	0.06	0.01	0.05	.96	
NFC × AAC	−0.20	0.06	−0.13	3.01	.01	
*Positive attitudes towards Gypsies*						
(Constant)	3.62	0.03		108.51	.01	.03
NFC	−0.10	0.03	−0.13	3.01	.01	
AAC	0.06	0.03	0.07	1.69	.91	
NFC × AAC	−0.07	0.04	−0.08	1.90	.06	
*Stereotype index*						
(Constant)	5.02	0.02		199.83	.01	.04
NFC	0.07	0.02	0.12	2.67	.01	
AAC	0.01	0.02	0.01	0.03	.97	
NFC × AAC	0.07	0.03	0.11	2.50	.01	

**Fig. 1:**
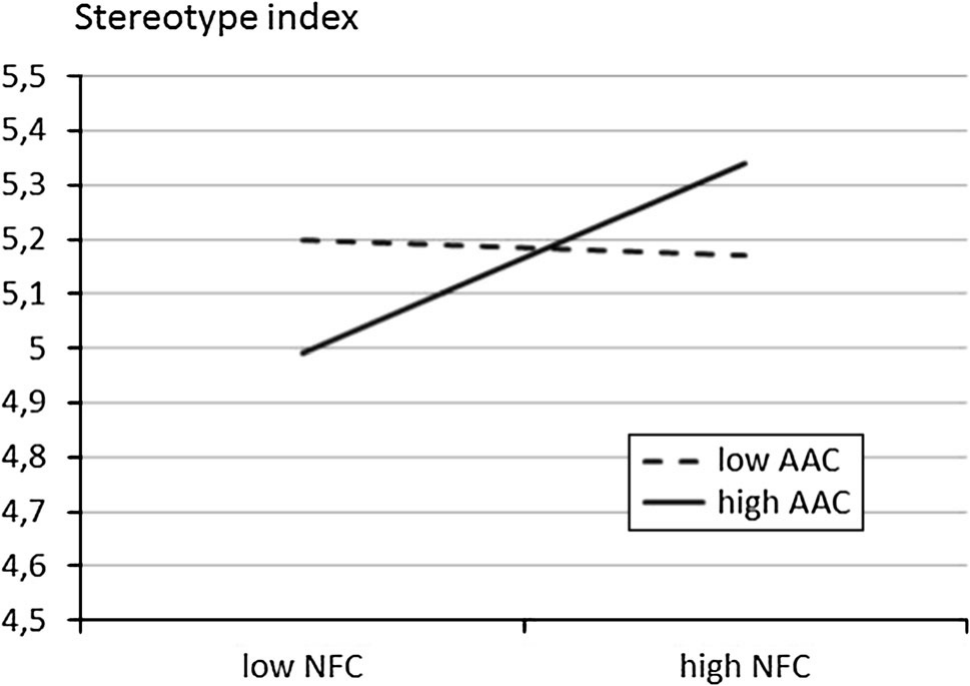
Regression lines showing the index of stereotype as a function of NFC, for low and high AAC participants (Study 1)

**Fig. 2:**
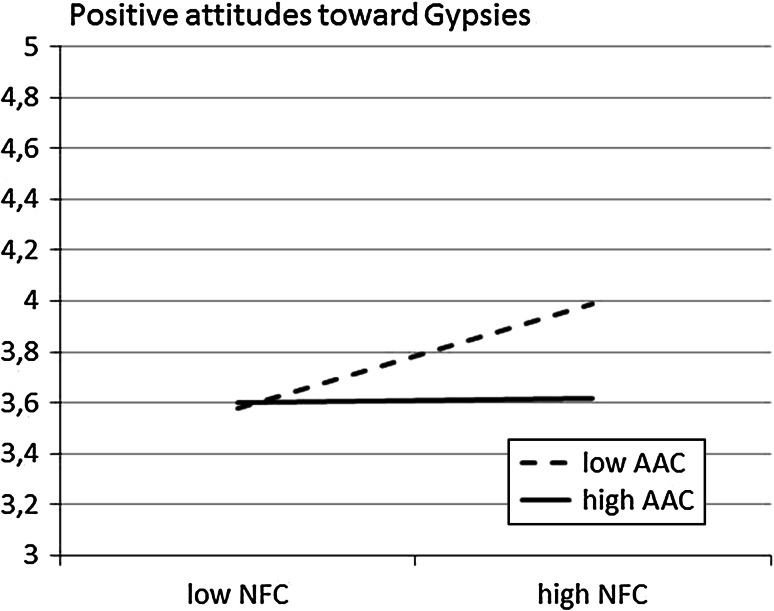
Regression lines showing the positive attitudes towards Gypsies as a function of NFC for low and high AAC participants (Study 1)

**Fig. 3:**
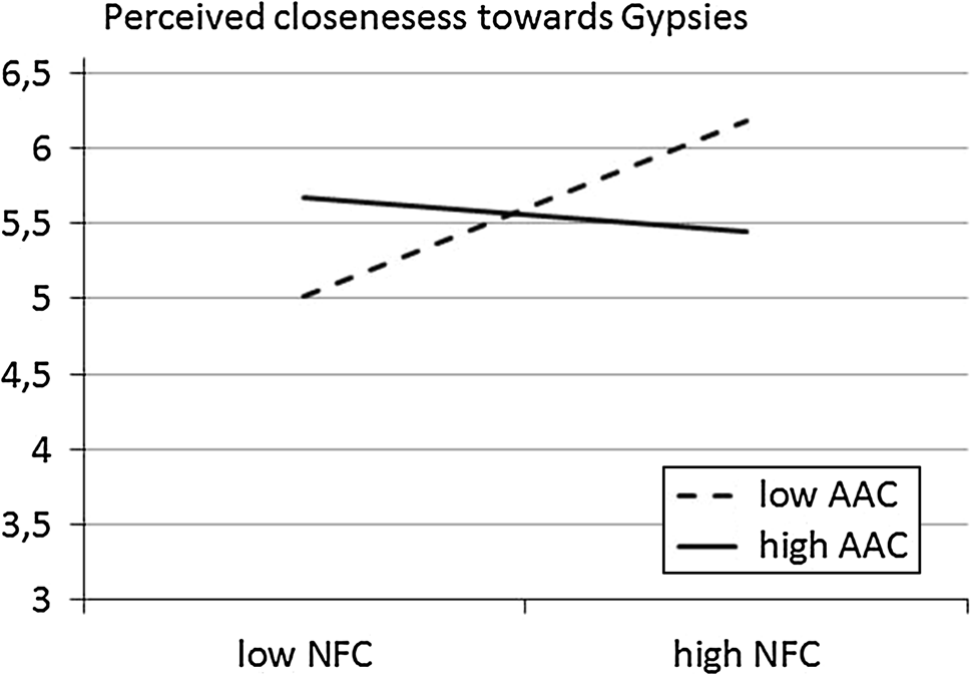
Regression lines showing the index of perceived closeness towards Gypsies as a function of NFC for low and high AAC participants (Study 1)

To illustrate the interactions, regression lines for dependent variables as a function of NFC were calculated separately for high and low AAC at 1 SD below and above the mean (representing the top and bottom percentiles; Cohen and Cohen 1983; Holmbeck 1997). Simple slope analysis indicated a positive association between NFC and the stereotype index for high-AAC participants (*b* = 0.14; *t*(519) = 3.78; *p* = .01), and a negative but non-significant association for low-AAC participants (*b* = −0.02, *t*(519) = 0.43, *p* = .66). There was also a positive association between NFC and positive attitudes for low-AAC participants (*b* = 0.26; *t*(519) = 3.21; *p* = .01) and a non-significant association for high-AAC participants (*b* = 0.01; *t*(519) = 0.14; *p* = .89). Similarly, the slope of NFC on perceived closeness for low-AAC respondents was positive and significant (*b* = 0.42, *t*(519) = 5.87, *p* = .01), but this slope was non-significant for high-AAC respondents (*b* = 0.02, *t*(519) = 0.03, *p* = .77). The interaction patterns are displayed in Figs. 1, 2 and 3.

### Discussion

These results demonstrated that the relationship between positive attitudes and NFC was influenced by AAC, when it was measured as an individual characteristic. Specifically, we found that a positive relationship between positive attitudes and NFC existed only when participants had a low level of AAC. It was hypothesised that among participants with a low AAC, NFC would reduce confidence in schematic beliefs and alter their initial opinions, which in turn, would increase their awareness of positive information about the stereotyped group, yielding a more balanced perception. As a result, the higher the NFC, the less the participants would use one-sided and unequivocal descriptions of Gypsies. These arguments appeared even stronger when we examined the interaction of NFC and AAC on stereotypes. As we expected, for high AAC, NFC associated with having negative stereotypes of Gypsies, but for low AAC, there was no association between NFC and stereotyping. Low AAC may have prevented participants with increased NFC from constructing schema and applying them effectively, and thus, these participants may perceive outgroups ambiguously, not in a schematic and negative way.

## Study 2

In Study 2, we investigated positive attitudes towards Gypsies as in Study 1, but we experimentally decreased participants’ AAC. Although past research has mainly focused on the AAC as dispositional measure, we propose that AAC can be influenced by situational factors. Factors such as situational perceptions of mental resources, positive mood, or being in a powerless position have been found to affect individuals’ AAC (Clarkson et al. 2010; Kossowska and Bar-Tal 2013b; Otten and Bar-Tal 2002). In the research reported here, we attempted to induce a sense of uncontrollability by applying a helplessness training procedure to reduce AAC. Thus, we hypothesised that under low AAC, NFC would lead to positive attitudes towards Gypsies. We did not expect a relationship between NFC and positive attitudes toward Gypsies in the control group.

### Method

#### Participants

Fifty-four students from one of the universities in Krakow (40 women and 14 men; *M*_age_ = 21.00, SD = 1.43) took part in the study on a voluntarily basis, and they received credit points for their participation.

#### Materials and procedure

Participants were asked to take part in a study on impression formation. They were randomly assigned to one of the experimental conditions (helplessness and control). They received booklets containing demographic questions and the NFC scale, and then they completed the experimental tasks and a questionnaire measuring attitudes towards Gypsies. Later, they were debriefed and thanked.

We used the 32 item version of the NFC scale (Webster and Kruglanski 1994; Polish version: Kossowska 2003), excluding the decisiveness subscale. The Cronbach’s *α* was .85 (*M* = 3.54; SD = 0.42). The perceived AAC of the participants was manipulated using a computerised version of a concept formation task, which was originally developed as the Informational Helplessness Training procedure (Sedek and Kofta 1990). This task was used with success by Otten and Bar-Tal (2002) to manipulate AAC. In the helplessness inducing condition (low AAC), participants were requested to complete six tasks that were, in fact, unsolvable. In the control condition, participants were given solvable tasks. The tasks consisted of six discrimination problems with eight trials for each problem. Participants had to identify a critical feature that defined a series of eight figures. Figures varied in four ways, with each having two features: (a) size (small or large), (b) shape (triangle or circle), (c) surface (plain or striped), and (d) capitalisation of the letter ‘r’ in the middle of a figure (r or R). For each discrimination problem, the computer programme consecutively presented the eight figures, each of which was labelled, ‘yes, feature is included’ or ‘no, feature is not included’. After being exposed to the eight figures, each participant was given a full list of possible solutions (i.e., the list of eight figure features). The task was explained and demonstrated in an example in which the task was solvable. The actual manipulation began after ensuring that participants understood the nature of the task.

In the low AAC condition, the eight figures in each discrimination problem were shown consecutively to participants. The participants were not told about the correctness of their responses under the pretext that they would be given this information after completing the study. This procedure has been shown to produce a state of irreducible uncertainty, which can be considered a reliable predictor of self-reported cognitive difficulties with thinking and attention (Kofta and Sedek 1999). In the control group, participants performed solvable discrimination tasks. Participants were not informed about their success or failure after completing the six problems in either of the two conditions.

Following the task, participants were requested to mark the target feature and to answer three questions on six-point scales: (a) How certain are you that you properly guessed the target feature? (1 = uncertain 6 = I am absolutely certain), (b) How did the problem solving go? (1 = it was very easy, 6 = it was very hard), and (c) How confident do you feel that you can solve a subsequent problem (1 = I am certain I will not succeed, 6 = I am certain I will succeed). The mean of the three questions (after recoding the second question) served as a manipulation check of the AAC (Cronbach’s *α* = .89).

To measure positive attitudes towards Gypsies, we used four items: “As viewed by society, do Gypsies make your group members tend to: help, protect, cooperate with, and include them into the group?” This measure was recommended by Cuddy et al. (2008) as a way to elicit cultural beliefs while minimising social desirability. The scale ranged from 1 (strongly disagree) to 7 (strongly agree). From these assessments, we calculated a mean score for positive attitudes towards Gypsies (Cronbach’s α = .72). We decided to use different measures of positive attitude than those used in Study 1 because we were interested in being able to generalize the results to other popular measures of attitude. In addition, in previous study, the positive behaviors measure correlates positively and highly with the allophilia and the closeness scales (see also Pittinsky et al. 2011a). Moreover, attitude theories posit that the affective, cognitive, and behavioural correlates of evaluation tend to converge (e.g., Frijda 1986; Scherer 1988; Smith and Ellsworth 1985), and that situations and their corresponding cognitive appraisals elicit discrete patterns of emotions, which, in turn, trigger specific behavioural responses (e.g., offensive action) (Frijda et al. 1989; Izard 1991; Izard et al. 1993). Moreover, we wanted to measure the aspect of attitudes related to behavioral intentions because they are less spontaneous than just mere evaluations and require the application of schematic knowledge to a greater extent. Thus, it was reasonable to expect the similar effect of NFC and AAC not only on the allophilia and closeness scales (affective component), but also on the scale measuring behavioural tendencies.

### Results

To test whether AAC had been successfully decreased, the answers to the three questions measuring AAC after each of the six problem-solving tasks were averaged. Lower average scores were found among participants in the helpless condition compared to the control group (*t*(54) = 10.82, *p* = .01). The means for the helpless condition and the control condition, respectively, were: *M* = 1.96, SD = 0.75 and *M* = 5.36, SD = 1.19. Thus, we may conclude that our manipulation effectively induced a sense of inability to achieve closure.

As in Study 1, the gender of the participants did not significantly predict any outcomes, and therefore gender was excluded from the analyses. NFC was not significantly correlated with the condition variable or with positive attitudes towards Gypsies.

To examine the moderating effect of condition, two-step hierarchical regression analysis was performed on positive attitudes towards Gypsies. Experimental condition (dummy coded-1 for control/1 for low AAC) and NFC were entered into the regression equation in the first step. The interaction term (NFC × condition) was added in the second step. NFC was centred before the respective cross-products were computed. We found a significant effect of condition (*R* ^2^ = .08; *F*(1,50) = 4.57; *p* = .04) that demonstrated less positive attitudes towards Gypsies among participants in the helpless condition, in which AAC was experimentally decreased. Most importantly, the results of the analysis revealed a significant interaction between NFC and experimental conditions for positive attitudes towards Gypsies (*R* ^2^ = .17; *F*(1,50) = 5.63; *p* = .02; see Table 2).

**Table 2:** Regression coefficients (Study 2)

Dependent variables	Unstandardised coefficients		Standardised coefficients	t	Sig.	Adj. *R* ^2^
	B	SE	Beta			
*Positive attitudes towards Gypsies*						
(Constant)	3.47	0.16		21.15	.01	.09
NFC	0.25	0.20	0.18	1.28	.20	
Condition	−0.39	0.16	−0.30	2.39	.02	
NFC × condition	0.47	0.20	0.32	2.37	.02	

Condition coded −1 control/1—low AAC

**Fig. 4:**
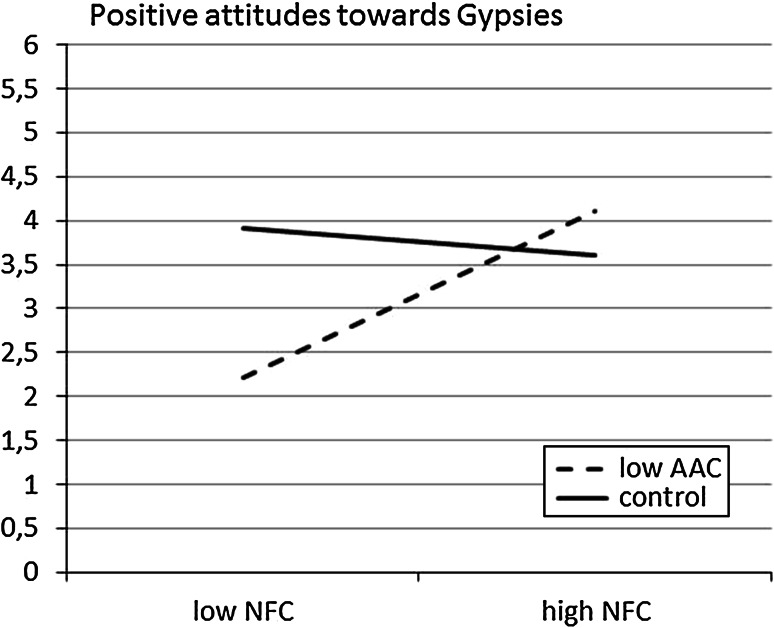
Regression lines showing the index of positive attitudes (i.e., behavioural tendencies) towards Gypsies as a function of NFC for low AAC and control group (Study 2)

To illustrate the pattern of the results, the regression lines for NFC that predicted the dependent variable were calculated separately for the low AAC and control conditions. Simple slopes analysis indicated that NFC: had a significant, positive association with positive attitudes towards Gypsies for the low AAC participants (*b* = 1.48; *t*(54) = 2.17; *p* = .04); and, had a non-significant, negative association with positive attitudes towards Gypsies for the control group (*b* = −0.45; *t*(54) = 1.01; *p* = .32). See Fig. 4.

### Discussion

In Study 2, we demonstrated that NFC is related to positive attitudes toward Gypsies among people with a decreased sense of personal control (i.e., low AAC condition) compared to a control group without a decreased sense of personal control. It can be argued that in such motivational settings people cannot effectively construct and apply dominant cognitive schemas (dispositional or situationally activated), and instead they develop more non-schematic knowledge structures (see Kofta et al. 2011). In the case of our study, the findings contribute to the existing literature by showing a similar pattern of results in the domain of attitudes. That is, in people who experience lower levels of AAC, NFC is linked to positive attitudes towards the outgroup, in contrast to people from a group without personal control deprivation. This seemingly paradoxical effect of increased positivity towards negatively evaluated outgroups reflects the specific role of NFC when one’s experience of AAC is low. In such cases, the commonly observed pattern of increased negativity towards outgroups reverses, because one’s initial confidence in one’s own knowledge and cognitive abilities is undermined. It seems that situations that decrease personal control and the perceived ability to obtain certain knowledge, yield less uniform evaluations of outgroup. This leads to higher positive ratings of negatively evaluated outgroup, as we have shown in this study. It is worth stressing that these results corroborate previous findings (e.g., Kruglanski et al. 2002; Otten and Bar-Tal 2002).

## Summary and concluding discussion

Our studies demonstrated that for participants with low AAC, there was a positive relationship between NFC and positive attitudes towards a negatively stereotyped outgroup. We suggest that in the low AAC condition, NFC may not have been related to the construction and effective use of schemas (i.e., negative stereotype of Gypsies in this research), and this may have led to systematic processing information that is inconsistent with stereotypes (i.e., positive). As a result, the higher the NFC, the less the participants tended to use one-sided and unequivocal descriptions of Gypsies, thus making it easier to foster positive attitudes towards the outgroup. We demonstrated this effect when using three different measures of positive attitudes toward Gypsies, which leads us to think that this is a general phenomenon that is not dependent on the way that positive attitudes are measured.

It is worth stressing that we did not expect the same mechanism to influence negative attitudes. Although we did not investigate this in the current research, we expect that NFC is related to negative attitudes towards outgroups among people with high, but not low AAC. Experiencing high AAC, people might expect that they can easily reduce uncertainty by applying schematic knowledge, and when that information is negative (i.e., negative stereotype), they use it to foster negative attitudes towards outgroups. We believe that our findings are in line with research that suggests that positive and negative attitudes should be treated as separate dimensions (e.g., Cacioppo and Berntson 1994; Pittinsky et al. 2011b).

Moreover, our results corroborate the motivation-cognitive model of information processing presented by Bar-Tal et al. (1997), which extends the lay epistemic theory (Kruglanski 1989). One of the implicit assumptions of the lay epistemic theory is that because the cognitive processes associated with high NFC are relatively effortless and automatic, achieving closure is an easy default option, which is available regardless of an individual’s ability to carry it through. However, our results provide evidence for the idea that the construction and application of cognitive schemas is not relatively automatic and effort-free, and thus may not always be the most efficient means of achieving certainty. Specific ability (i.e., AAC) is apparently required for an individual to realise his/her motivation to achieve certainty by reaching closure. Our findings suggest that inclusion of the concept of AAC as an inherent component of the widely accepted theory of lay epistemology may elucidate our understanding of the process of knowledge formation and use.

It is also important to note that this research demonstrated that AAC could be situationally lowered. The validity of our manipulation of AAC as a situational variable was supported by our manipulation checks and it has been corroborated in studies by Bar-Tal et al. (e.g., 1999; Bar-Tal and Guinote 2002; Kossowska and Bar-Tal 2013a), who found comparable NFC × AAC interaction effects based on individual-characteristic variables. Importantly, we demonstrated that a decreased sense of control might, in fact, decrease reliance on accessible constructs, regardless of their chronic or temporal accessibility. So far, it has been shown when people have a low sense of control, they appear to be less inclined to rely on stereotypes and schematic knowledge to obtain more specific, individual information from the environment (Kofta and Narkiewicz-Jodko 2003). In a similar vein, when AAC is low, people with increased NFC do not rely on easily accessible schemas, such as stereotypes (Bar-Tal and Guinote 2002). Similar results have been reported for the effect of self-anchoring in the minimal group paradigm. That is, experimentally decreased levels of AAC impeded the tendency to rely on the self as a heuristic for ingroup evaluations (Otten and Bar-Tal 2002). Our research shows that for people with decreased levels of personal control and perceived AAC, NFC can also lead to the expression of more positive intergroup attitudes. We believe that this is an important contribution to research on the malleability of stereotypes and attitudes, which has shown that many motivational (e.g., understanding, controlling, self-enhancement etc.) and cognitive (e.g., focus of attention, configuration of stimulus cues etc.) factors can change individual’s perception and evaluation of outgroup members (Blair 2002; Fiske 2003; Kunda and Spencer 2003). Additionally, we would argue that it is important to take into account the interaction between situationally activated motives (e.g., uncertainty reduction) and personal predispositions (e.g., perceived ability or self-efficacy in dealing with uncertainty) when analysing conditions that promote belief or attitude change.

One could, however, argue that AAC (both as manipulated and as measured) could be seen as a proxy for fear of invalidity, which was defined by Kruglanski (1989) as a concern with error or the consequences of a decision. This concern is associated with the continued generation and evaluation of information relevant to a given decision (see Kruglanski and Freund 1983). Such an increase in the fear of invalidity, may be expected to lower the NFC, resulting in less negative attitudes toward the stereotyped group. Moreover, one might argue that the stereotype attenuating influence of the AAC is more apparent when NFC is high because there is greater room for NFC reduction. Though high NFC and fear of invalidity often co-occur, and thus may be lumped together (see Kruglanski et al. 1997), previous research shows that NFC is unrelated to AAC (Bar-Tal and Kossowska 2010). Also, the results of Study 2, in which AAC was experimentally decreased, are not consistent with this expectation. In fact we found that among low AAC participants, NFC is positively related to positive attitudes. Although in light of our study we think it is improbable that these results are attributable to fear of invalidity, future research is needed to address this issue in full.

Finally, it is worth mentioning some important limitations of our studies. Pittinsky et al. (2011b) suggested that positive and negative attitudes towards outgroups are distinct constructs, and that both of them should be measured to understand behaviours towards those groups. In the current research, however, we focused on positive attitudes only. As mentioned above, we did not predict a significant relationship between NFC and positive attitudes for high AAC individuals, and we did not find such a relationship. Future research should measure both negative and positive attitudes at the same time to fully study the NFC × AAC interaction model. We did not expect that NFC would be negatively related to positive attitudes among high AAC participants: in Study 1 we measured AAC, and in Study 2 we lowered AAC. We assumed that NFC is related to perceiving outgroups as homogeneous (Dijksterhuis et al. 1996). Thus, members of negatively perceived groups should be evaluated more negatively by people with increased NFC, and high AAC will increase this relationship. Although the results indicate that high AAC increased the use of stereotypes when NFC is higher, there were no significant relationship between NFC and stereotyping for low AAC participants. This result may be interpreted to mean that in low AAC participants, NFC is related to problems with constructing and applying schemas efficiently.

In future research, therefore, it would be important to measure negative and positive components at the same time by using a broader range of attitudinal measures. To further test our hypotheses, we would need to measure the ambiguity of people’s outgroup evaluations and the variability of their outgroup perceptions. Based on the “ability × need” model of knowledge formation, we would expect ambiguity and variability to increase with NFC in low ability conditions. Additionally, different groups could be used as the targets of attitudes; these could be groups that are evaluated in a less uniformly negative fashion, or even groups who are positively evaluated, to replicate the decrease in positivity under lowered perception of ability. Last, but not least, it is important to take the intergroup perspective into account, and to consider such variables in the evaluation of outgroups, e.g., social status. It might be expected that there would be a stronger increase in positive attitude towards low versus high status outgroups when one’s perceived ability to achieve closure is high but the epistemic motivation remains strong.

## Footnotes

1 The survey was built using SurveyMonkey^®^.

2 The stereotypical characteristics used in the study: unreliable, educated, lazy, friendly, competent, moral, dishonest, family man, orderly, neat, intrusive, insolent, filthy. Positive characteristics were reversed before calculation the index of negative stereotype.
